# Supplementary figures and images for: Combining HF rTMS over the Left DLPFC with Concurrent Cognitive Activity for the Offline Modulation of Working Memory in Healthy Volunteers: A Proof-of-Concept Study
Source: Brain Sci. 2020 Feb 4;10(2):83. doi: 10.3390/brainsci10020083 (PMC7071618; doi:10.3390/brainsci10020083)

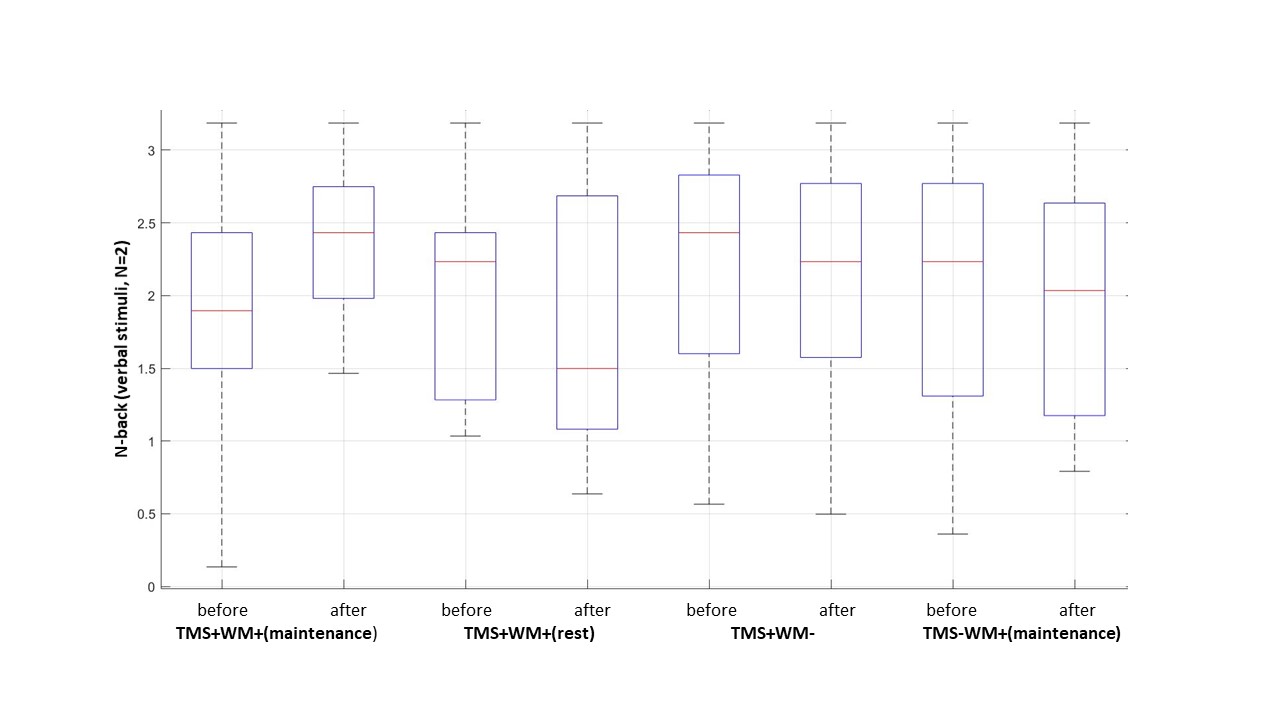

Supplement: Supplementary file 1 [file brainsci-10-00083-s001.zip › Supplementary Figures/Figure S1.JPG]

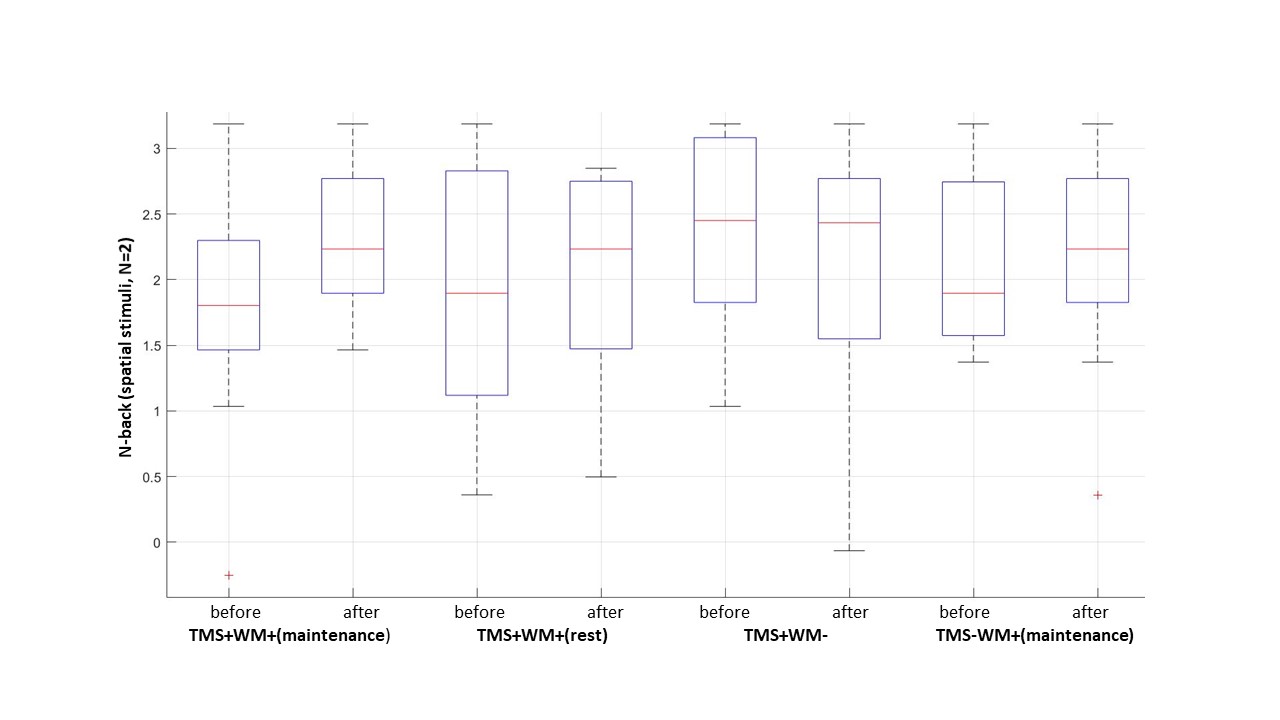

Supplement: Supplementary file 1 [file brainsci-10-00083-s001.zip › Supplementary Figures/Figure S2.JPG]

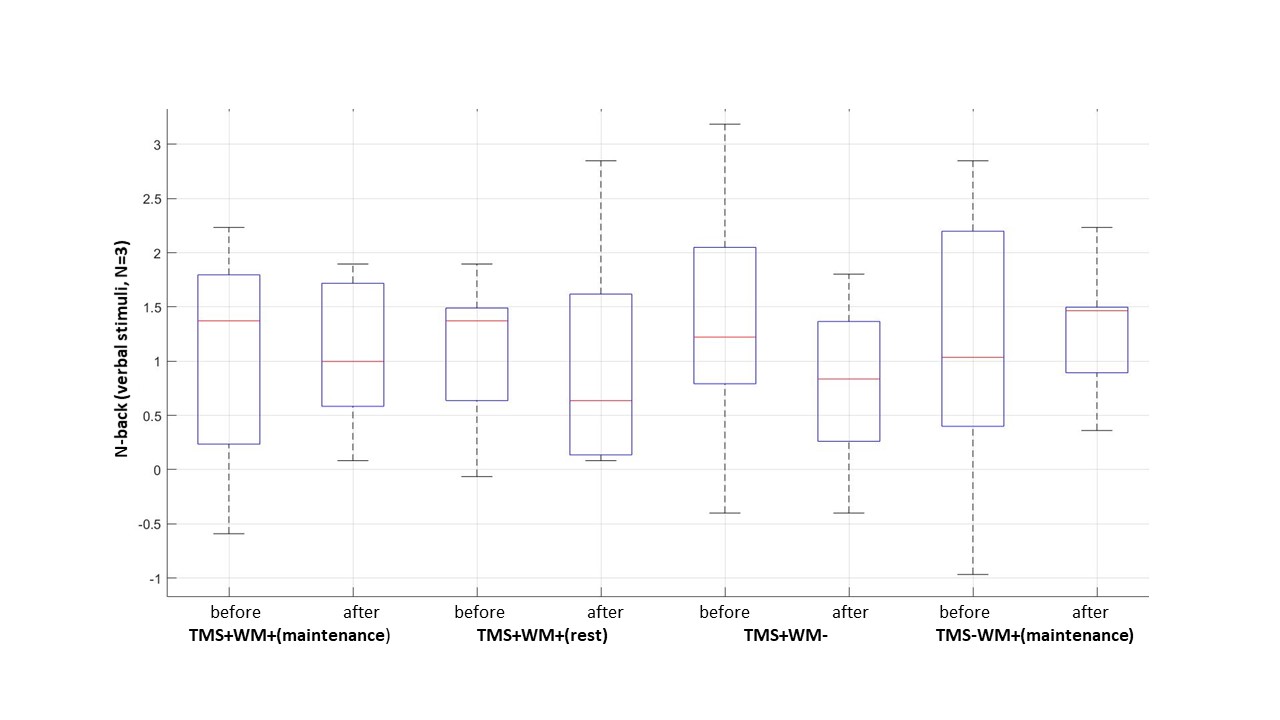

Supplement: Supplementary file 1 [file brainsci-10-00083-s001.zip › Supplementary Figures/Figure S3.JPG]

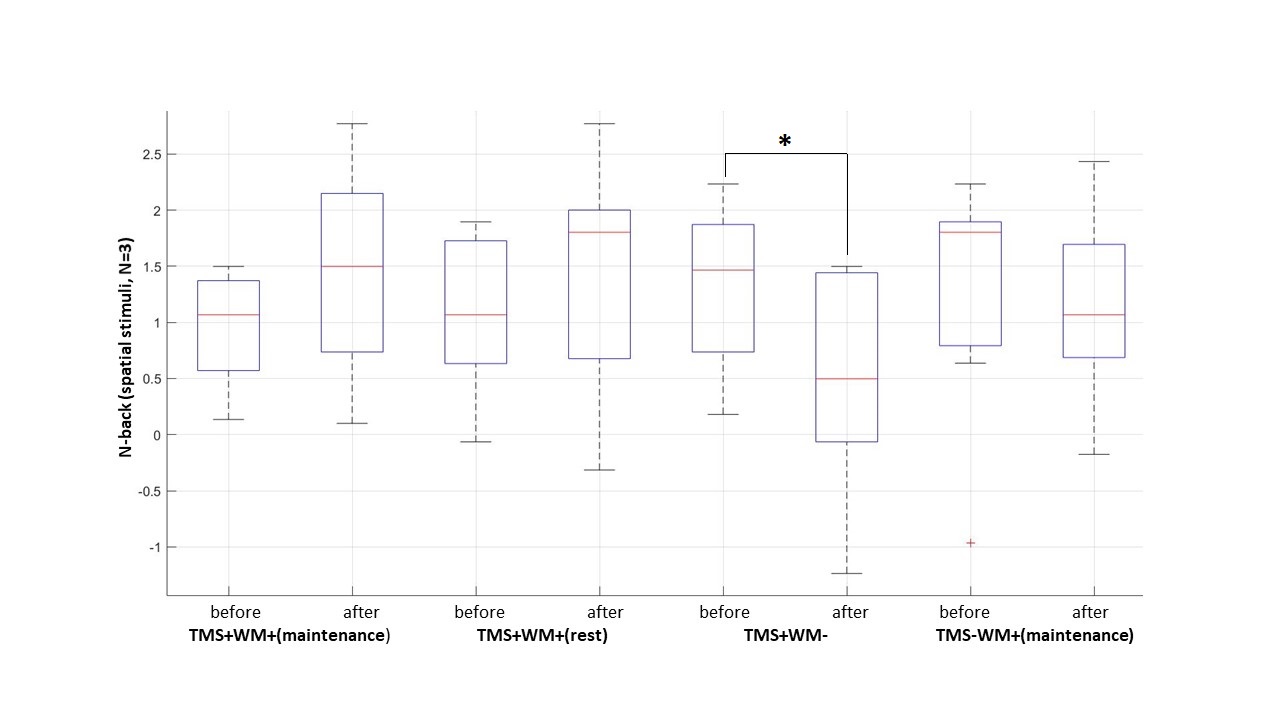

Supplement: Supplementary file 1 [file brainsci-10-00083-s001.zip › Supplementary Figures/Figure S4.JPG]

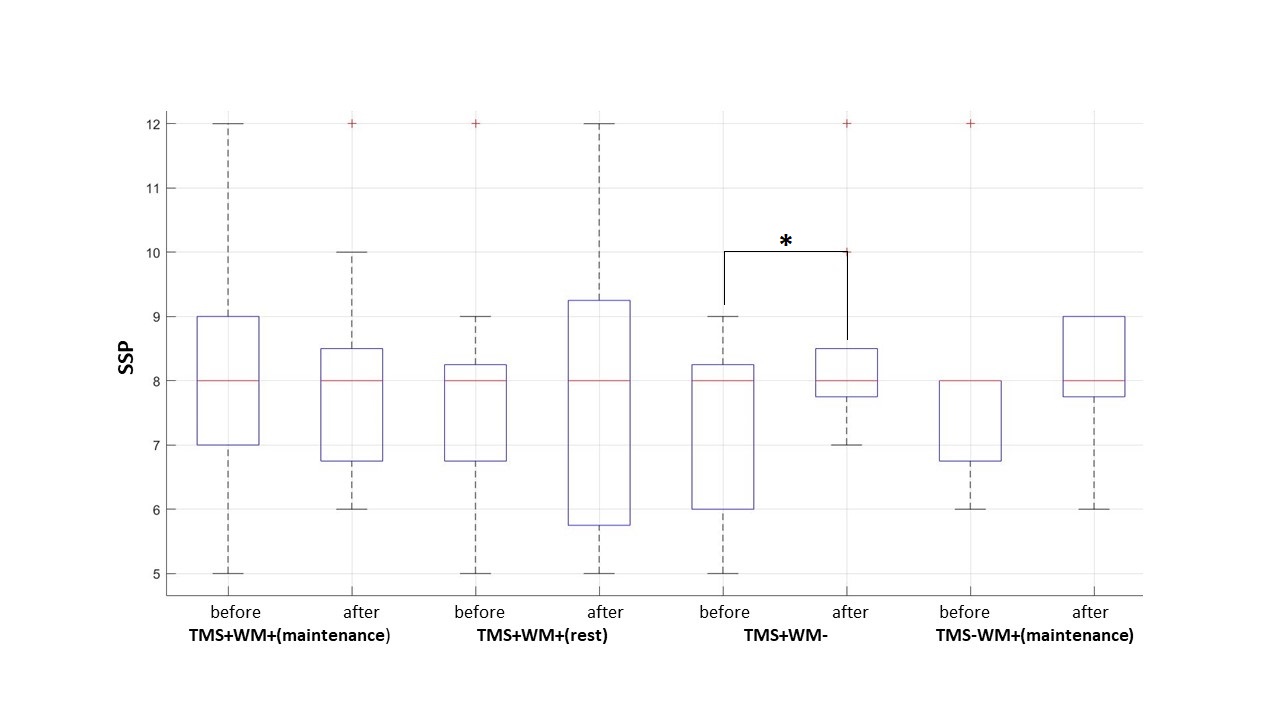

Supplement: Supplementary file 1 [file brainsci-10-00083-s001.zip › Supplementary Figures/Figure S5.JPG]
